# Experiments on the Mass of Blood Relative to the Weight of the Body

**Published:** 1845-06

**Authors:** 


*Experiments on the Mass of Blood relative to the Weight of the Body.* By M. Wanner.—As it was impossible to ascertain in man the important fact of the weight of the blood to that of the body, M. Wanner made several experiments on the lower animals. The animals were first carefully weighed, and then bled to death by the butchers, and the blood weighed.

A bullock, weighing 1650 lbs. imperial, yielded 60 lbs. of blood. The proportion was therefore as 1 to 23·81, or rather more than 4 per cent. of blood.

Another bullock, weighing 1640 lbs., yielded 65 lbs. of blood. The proportion was therefore almost the same as the first, 1 in 23.73.

A cow, weighing 1293 imperial lbs., yielded 59 lbs. of blood. The proportion was therefore 1 in 21·77, or nearly 5 per cent. of blood.

A sheep, weighing 110 lbs., yielded 5½ lbs. of blood, or in the proportion of 1 to 22·72, or about 4½ per cent. of blood.

Another sheep, weighing 88 lbs., yielded 4 lbs. of blood, or in the proportion of 1 in 20, just 5 per cent. of blood.

In a rabbit the proportion of blood was as 1 to 25 exactly.

As it may be safely inferred that man follows the same laws in this respect as the lower animals, it may be deduced that a person weighing 100 lbs. has 5 lbs. of blood in his body,—a person weighing 200 lbs. nearly 10 lbs. of blood, and so on. The practical conclusion which the author deduces from this fact is, that a blood-letting to the extent of two cups from a woman weighing 100 lbs. is as much as one of four cups from a man weighing 200 lbs. The blood-letting ought, therefore, in every case to be proportioned to the weight of the individual. Thus, if 2 lbs. of blood were drawn from an individual 100 lbs. in weight, it would abstract nearly one-half of the blood from the body. Nine leeches, each abstracting half an ounce, would draw half of the blood from a child 5 years of age, whose average weight is about 30 lbs., and as 4 or 5 ounces is all the available blood in a child at birth, this fact should make us cautious in allowing blood to escape from the chord in cases of apoplexy, &c.

It is remarked by M. Guerin that these experiments merely relate to the available blood in the body, and though true in a physiological and practical, are not so in a pathological sense.—*Ibid from Ibid.*

